# Cardiovascular complications of diabetes: role of non-coding RNAs in the crosstalk between immune and cardiovascular systems

**DOI:** 10.1186/s12933-023-01842-3

**Published:** 2023-05-24

**Authors:** Gaia Spinetti, Martina Mutoli, Simona Greco, Federica Riccio, Soumaya Ben-Aicha, Franziska Kenneweg, Amela Jusic, David de Gonzalo-Calvo, Anne Yaël Nossent, Susana Novella, Georgios Kararigas, Thomas Thum, Costanza Emanueli, Yvan Devaux, Fabio Martelli

**Affiliations:** 1 Laboratory of Cardiovascular Pathophysiology and Regenerative Medicine, IRCCS MultiMedica, Milan, Italy; 2 Molecular Cardiology Laboratory, IRCCS Policlinico San Donato, Milan, Italy; 3 National Heart & Lung Institute, Imperial College London, London, UK; 4 Institute of Molecular and Translational Therapeutic Strategies (IMTTS), Hannover Medical School, Hannover, Germany; 5 HAYA Therapeutics SA, Lausanne, Switzerland; 6 Translational Research in Respiratory Medicine, University Hospital Arnau de Vilanova and Santa Maria, IRBLleida, Lleida, Spain; 7 CIBER of Respiratory Diseases (CIBERES), Institute of Health Carlos III, Madrid, Spain; 8 Department of Surgery, Leiden University Medical Center, Leiden, the Netherlands; 9 Department of Physiology, University of Valencia - INCLIVA Biomedical Research Institute, Valencia, Spain; 10 Department of Physiology, Faculty of Medicine, University of Iceland, Reykjavík, Iceland; 11 Cardiovascular Research Unit, Department of Precision Health, Luxembourg Institute of Health, Strassen, Luxembourg

**Keywords:** Diabetes, Cardiovascular diseases, Non-coding RNAs, Inflammation

## Abstract

Diabetes mellitus, a group of metabolic disorders characterized by high levels of blood glucose caused by insulin defect or impairment, is a major risk factor for cardiovascular diseases and related mortality. Patients with diabetes experience a state of chronic or intermittent hyperglycemia resulting in damage to the vasculature, leading to micro- and macro-vascular diseases. These conditions are associated with low-grade chronic inflammation and accelerated atherosclerosis. Several classes of leukocytes have been implicated in diabetic cardiovascular impairment. Although the molecular pathways through which diabetes elicits an inflammatory response have attracted significant attention, how they contribute to altering cardiovascular homeostasis is still incompletely understood. In this respect, non-coding RNAs (ncRNAs) are a still largely under-investigated class of transcripts that may play a fundamental role. This review article gathers the current knowledge on the function of ncRNAs in the crosstalk between immune and cardiovascular cells in the context of diabetic complications, highlighting the influence of biological sex in such mechanisms and exploring the potential role of ncRNAs as biomarkers and targets for treatments. The discussion closes by offering an overview of the ncRNAs involved in the increased cardiovascular risk suffered by patients with diabetes facing Sars-CoV-2 infection.

**Graphical Abstract:**

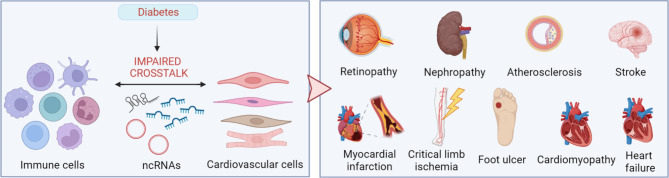

## Introduction

**Fig. 1:**
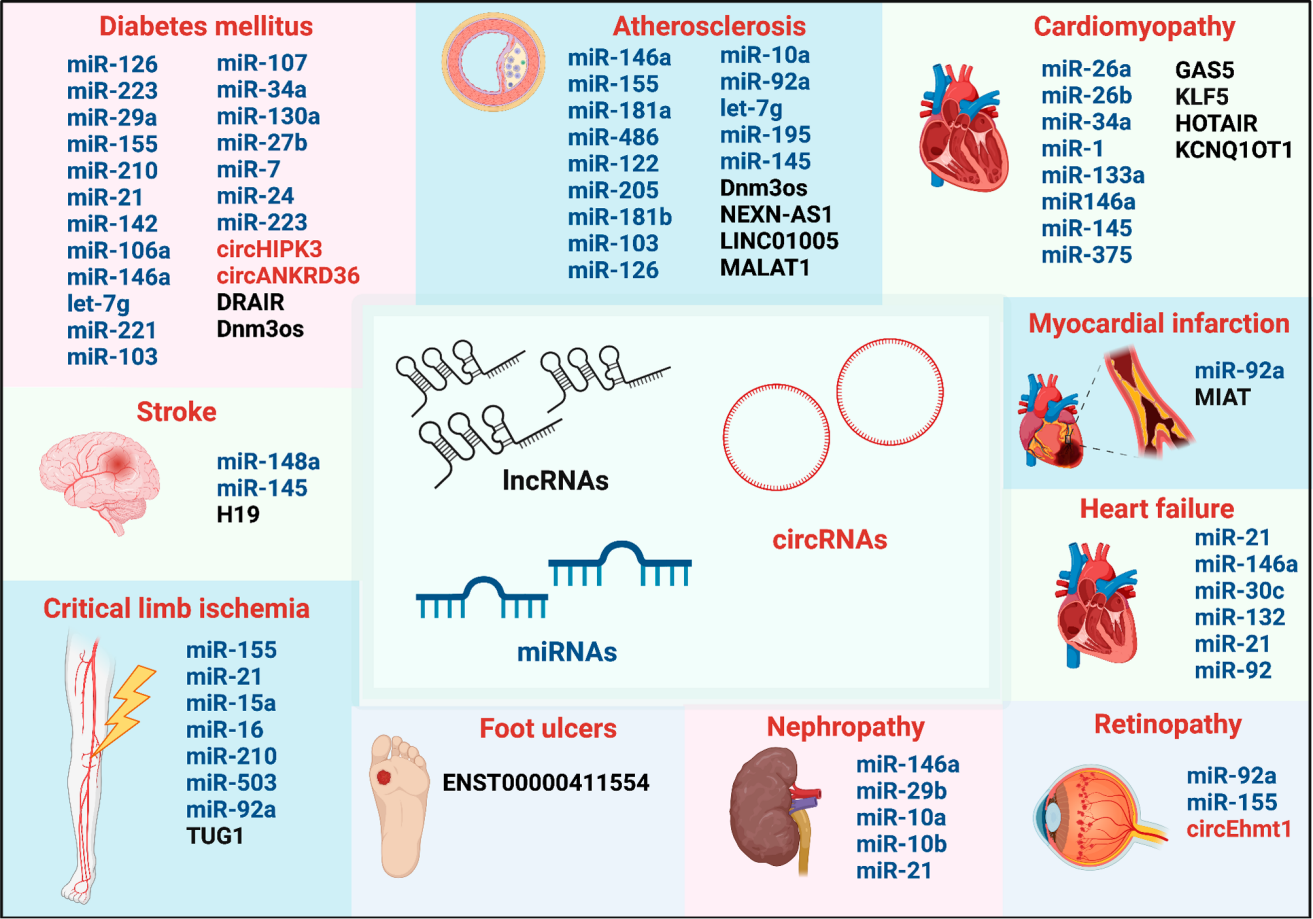
ncRNAs involved in DM and its cardiovascular complications. The diagram summarizes lncRNAs (black), circRNAs (red) and miRNAs (blue) involved in DM and its related cardiovascular complications i.e. atherosclerosis, cardiomyopathy, MI, HF, retinopathy, nephropathy, foot ulcers, CLI and stroke. DM: diabetes mellitus, MI: myocardial infarction, HF: heart failure, CLI: critical limb ischemia. Figure created with BioRender.com online tool

Diabetes mellitus (DM) is an important cause of functional impairment, morbidity, and mortality, representing a heavy economic burden for global healthcare systems. About 537 million adults (20–79 years) were estimated to be living with DM worldwide in 2021, and most of them were affected by Type 2 DM (T2DM). This number has been predicted to rise to 643 million by 2030 and to 783 million by 2045 [1]. Notably, DM-related mortality and reduction in life expectancy are greater in women than men [2, 3]. DM is characterized by high levels of blood glucose caused by impairment in the insulin production and/or signalling. Hyperglycemia, directly and indirectly, alters cells of the cardiovascular system leading to microvascular and macrovascular diseases in a sex-dependent manner. In particular, microvascular complications include diabetic retinopathy and nephropathy while macrovascular complications mainly refer to accelerated atherosclerosis in different areas including cerebrovascular atherosclerotic disease manifesting as stroke, coronary artery disease (CAD) that can result in an acute myocardial infarction (MI) and peripheral arterial disease (PAD) that can evolve into critical limb ischemia (CLI), resulting in wound healing inhibition and diabetic foot ulcers. Other macrovascular complications refer to cardiac dysfunction, including cardiomyopathy and heart failure (HF) [4–9]. A state of low-grade inflammation characterizes both DM and atherosclerosis together with a decrease/impairment in cardiovascular protective bone marrow (BM)-derived hematopoietic stem/progenitor cells (HSPCs) and a pro-inflammatory shift of mature immune cells, resulting in the impairment of the repair processes needed to maintain tissue homeostasis [10–14]. Different classes of leukocytes belonging to both innate and adaptive immunity, e.g. monocytes, macrophages, dendritic cells (DCs), T-lymphocytes, B-lymphocytes, and natural killer (NK) cells regulate the DM-associated inflammatory response, in a time- and tissue-specific manner [15]. However, the molecular pathways through which DM elicits an immune response and how this contributes to altering cardiovascular homeostasis have yet to be fully characterized. Hence, understanding the complex mechanisms of communication between immune and cardiovascular cells is pivotal for the identification of novel therapeutic targets and potential biomarkers for disease prognosis. In this respect, non-coding RNAs (ncRNAs) represent a still insufficiently studied class of transcripts that may play a fundamental role. Indeed, ncRNAs are major regulators and players of both cardiovascular and immune functions in DM and its related cardiovascular complications (Fig. 1) [16–18]. Here we discuss the roles of ncRNAs in the crosstalks between immune and cardiovascular cells underlying the onset and perpetuation of DM and its related cardiovascular diseases (CVDs), highlighting the implication of aging and biological sex in such a contest. Furthermore, selected, key DM-modulated peripheral blood (PB) ncRNAs are suggested as early biomarkers of disease development and outcome, and, ultimately, as new therapeutic tools. The discussion closes by offering an overview of the ncRNAs involved in the increased cardiovascular risk suffered by patients with diabetes facing Sars-CoV-2 infection.

To identify relevant reports for this narrative review, we searched on PubMed the literature published in English up to March 2023, using as query: (“non-coding RNA” or “microRNA” or “long non-coding RNA”) and (“inflammation” or “immune system”) and (“cardiovascular”) and (“diabetes” or “diabetic”). We then excluded articles not dealing with DM, coronavirus disease 2019 (COVID-19), or immune and cardiovascular cells, and selected those providing the most robust information in terms of crosstalk between the two systems. Other articles were selected from the reference list of the retrieved publications.

## The ncRNAs world: definition and classes

The vast majority of the human genome is transcribed into RNA that is not translated into proteins. It is also well recognized that these ncRNAs play a central role in human physiology and pathology. However, so far, we have only started to scratch the surface of the collective function of the non-coding part of the genome. Novel types of RNA are still being discovered and novel functions identified. A very simplistic, operational, classification subdivides ncRNAs according to their size: ncRNAs shorter than 200 nucleotides are called small ncRNAs (sncRNAs) (Table 1), ncRNAs longer than 200 nucleotides are called long ncRNAs (lncRNAs) (Table 2) [19, 20]. Of all ncRNAs, microRNAs (miRNAs) are probably the most studied and best understood so far. miRNAs are sncRNAs, with a length of approximately 22 nucleotides. miRNAs are known to bind preferentially to the 3’ untranslated region of target messenger RNAs (mRNAs), thereby inducing either mRNA degradation or repression of translation, via the RNA-induced silencing complex. In general, a single miRNA has only a slight effect on the translation of an individual target, but as single miRNAs can target many different mRNAs, often in the same pathway or gene program, the effect of a single miRNA on cell and tissue function is significant. One mRNA can be regulated by more than one miRNA, which leads to the creation of highly-interconnected large signaling networks [21–23]. Moreover, variations in miRNA biogenesis generating alternative miRNA isoforms termed isomiRNAs and post-transcriptional modifications like A-to-I editing and methylation, which are altered under pathological conditions, form a new and exciting area in ncRNA research in the cardiovascular field [24–26].

**Table 1:** Small non-coding RNA classes

Reference	Type of small non-coding RNA	Length	Reported functions
[22]	miRNA	~ 22 nucleotides	- mRNA degradation - translational repression
[27]	piRNA	~ 24–31 nucleotides	- transposon repression
[28–31, 332]	snoRNA	60–200 nucleotides	- pseudouridylation or 2’O-ribose methylation of rRNAs, snRNAs, and tRNAs - mRNA methylation - alternative splicing and processing - mRNA stabilization
[37, 38]	tRF	16–35 nucleotides	- miRNA-like mRNA translational repression - enhance translation

**Table 2:** Long non-coding RNA classes

Reference	Type of long non-coding RNA	Length	Reported functions
[41, 42]	lncRNA	> 200 nucleotides	- repress/activate transcription - repress/enhance translation - impact on RNA stability
[43–45]	circRNA	> 200 nucleotides	- sponge miRNAs - protein binding - translation into polypeptides

Much less is known about other types of sncRNAs, like piwi-interacting RNAs (piRNAs), small nucleolar RNAs (snoRNAs), and transfer RNA (tRNA)-derived fragments (tRFs). piRNAs, typically 24–31 nucleotides long, are mostly expressed in germline cells, where they target and repress transposons. However, piRNAs are also found in somatic cells, where their function remains to be elucidated [27].

snoRNAs are principally part of the ‘housekeeping’ of the cellular machinery and consist of 60–200 nucleotides. They play a crucial role in processing and stabilizing ribosomal RNAs (rRNAs) and small nuclear RNAs (snRNAs), tRNAs *via* guiding important base modifications; H/ACA box snoRNAs guide pseudouridylation and C/D box snoRNAs guide 2’O-ribose methylation. However, many snoRNAs have no known rRNA or snRNA targets, which are referred to as ‘orphan’ snoRNAs and their function is mostly unknown [28, 29]. In addition, certain snoRNAs can also target and regulate mRNAs. For certain orphan C/D box snoRNAs, it has been shown that they can target mRNAs and guide mRNA methylation, direct 3’-end processing, alternative splicing, and mRNA stability [30, 31]. Of relevance, several snoRNAs gene clusters have been associated with both DM and CVDs [32–34].

As the name implies, tRFs are derived from either pre-tRNAs or mature tRNAs, and it has been only recently accepted that they are more than merely tRNA degradation products [35, 36]. Their length ranges from 16 to 35 nucleotides and they either enhance translation or have miRNA-like functions such as mRNA translational repression, but many other mechanisms of action likely remain to be uncovered [37, 38]. Importantly, tRFs are associated with cardiovascular events, such as stroke [39].

lncRNA is the collective term for all ncRNAs longer than 200 nucleotides. Roughly, lncRNAs are subdivided into two classes, namely linear lncRNAs and circular RNAs (circRNAs). The latter is produced by back-splicing of precursor coding and lncRNA transcripts [40]. A broad range of functions and mechanisms of action has been described for lncRNAs. Linear lncRNAs can act as molecular scaffolds or decoys, for example, having an effect on transcription (activating or repressing it), modifying chromatin structure, or guiding pre-mRNA processing, but they can also influence protein translation (enhancing or repressing it), or affect RNA turnover rates [41, 42]. CircRNAs are mostly known for their ability to ‘sponge’ miRNAs but may have other important functions such as interaction with RNA-binding proteins, directing their interaction with downstream target proteins or mRNAs. Moreover, a small subset of circRNAs is translated [43–46]. lncRNAs are involved in all forms of human physiology and pathology [47], but due to lower conservation, their study using animal models is methodologically more challenging.

## ncRNAs in the immune-cardiovascular cell interaction

Innate immune cells, i.e. neutrophils, monocytes/macrophages, NK, and DCs, are involved in the early steps of the inflammatory response and are associated with atherosclerotic diseases [13, 48]. In recent years, ncRNAs are emerging as activators or inhibitors of the innate inflammatory response due to their role in the regulation of inflammatory gene expression. Furthermore, they are involved in the differentiation and function of innate immune cells [49, 50]. Their altered expression in innate immune cells is linked to diabetic complications. These disorders display a chronic activation of pro-inflammatory pathways, as well as abnormal activation of innate and adaptive immunity. The diabetic environment strongly facilitates chronic inflammation of the endothelium, as it is characterized by high levels of low-density lipoproteins (LDLs), glucose, advanced glycation end-products, advanced lipoxidation end-products, and reactive oxygen species (ROS) [51–55]. Long-term exposure to the diabetic environment can damage the vascular endothelium by inducing post-translational modifications of biological molecules, which damage the endothelium and trigger the innate and adaptive immune system. Leucocytes contribute to the pro-oxidant and pro-inflammatory milieu by continuously infiltrating the endothelium, where they accumulate and are activated in an uncontrolled manner, producing pro-inflammatory molecules [56, 57]. Moreover, many studies reported an increased number of circulating leucocytes in T2DM [58, 59]. In the context of atherosclerosis, monocytes differentiate into macrophages, which internalize LDL thus forming foam cells. Both monocytes and macrophages are characterized by a pro-inflammatory phenotype in patients with DM [60, 61]. Inflammatory signaling causes an additional release of cytokines, vasoactive molecules, and proteases from macrophages and foam cells, generating an additional T-helper-1 (Th1) pro-inflammatory response with the release of pro-inflammatory cytokines, which contributes to local inflammation and plaque growth. Th1 cells have a pro-atherosclerotic function, unlike regulatory T cells (T-regs), which have a protective effect [62]. Later phases of the plaque’s development include local proteolysis, plaque rupture, and ultimately thrombus formation, all of which can result in life-threatening events, such as MI or stroke [8, 60]. It has been shown that the vascular wall of humans and mice include high levels of DCs [63, 64]. It is known that the DC subset can play pro- and anti-atherogenic roles at all phases of atherogenesis through their wide range of functions, including lipid uptake, efferocytosis, inflammation resolution, and antigen presentation [65]. Also, neutrophils contribute to the advancement of atherosclerosis by releasing neutrophils extracellular traps (NETs), which have been found in atherosclerotic plaques [66, 67]. NETs have been linked to plasmacytoid DC-driven autoimmune activation and the production of anti-double strand DNA antibodies, both of which have the potential to significantly worsen the development of atherosclerosis lesions [68, 69]. Recent evidence supports the role also for NK in atherosclerosis, as recently discussed [13]. These findings reveal how the diabetic milieu activates the immune system resulting in chronic inflammation and ultimately leading to the onset of atherosclerosis. How ncRNAs are intertwisted in these processes is the object of the following sections.

### Hematopoietic stem and progenitor cells

HSPCs contribute to the homeostasis of the cardiovascular system [70], especially the subpopulation of CD34 + cells [71]. Indeed, CD34 + HSPCs contribute to vessel repair and their function is known to be altered in DM [72]. Both mobilization of CD34 + HSPCs from BM and their angiogenic function are impaired in patients with DM, a condition often referred to as ‘mobilopathy’ at least in part associated with the deregulation of ncRNAs [10, 70, 73–76]. BM CD34 + HSPCs show an altered expression of miRNAs in DM, including miR-155 and miR-21 [14, 75] associated with lower cell survival and induction of apoptosis. A recent study from our group showed that, compared to healthy controls, BM-derived CD34 + HSPCs bear lower levels of miR-21 and higher levels of one of miR-21 targets, the tumor suppressor programmed cell death 4. Of note, these changes can be transferred to endothelial cells (ECs) via taurine upregulated gene 1, a lncRNA responsible for the sequestration of miR-21, delivering a death signal to ECs [77]. In addition, serum and PB circulating HSPCs from diabetic patients with CLI contain increased levels of miR-15a and miR-16 with consequential impairment in HSPCs migration and adhesion capabilities [78]. On the other hand, miR-210 overexpression improves HSPC hypoxia-induced, SDF-1-driven migration [79]. Interestingly, restoring the expression levels of miR-92a in CD34 + cells from patients with diabetic retinopathy has been associated with an anti-inflammatory effect [80]. Lastly, angiogenic miR-126 is modulated in DM in CD34 + cells and its transfer can impact the endothelium [81].

### Neutrophils

**Fig. 2:**
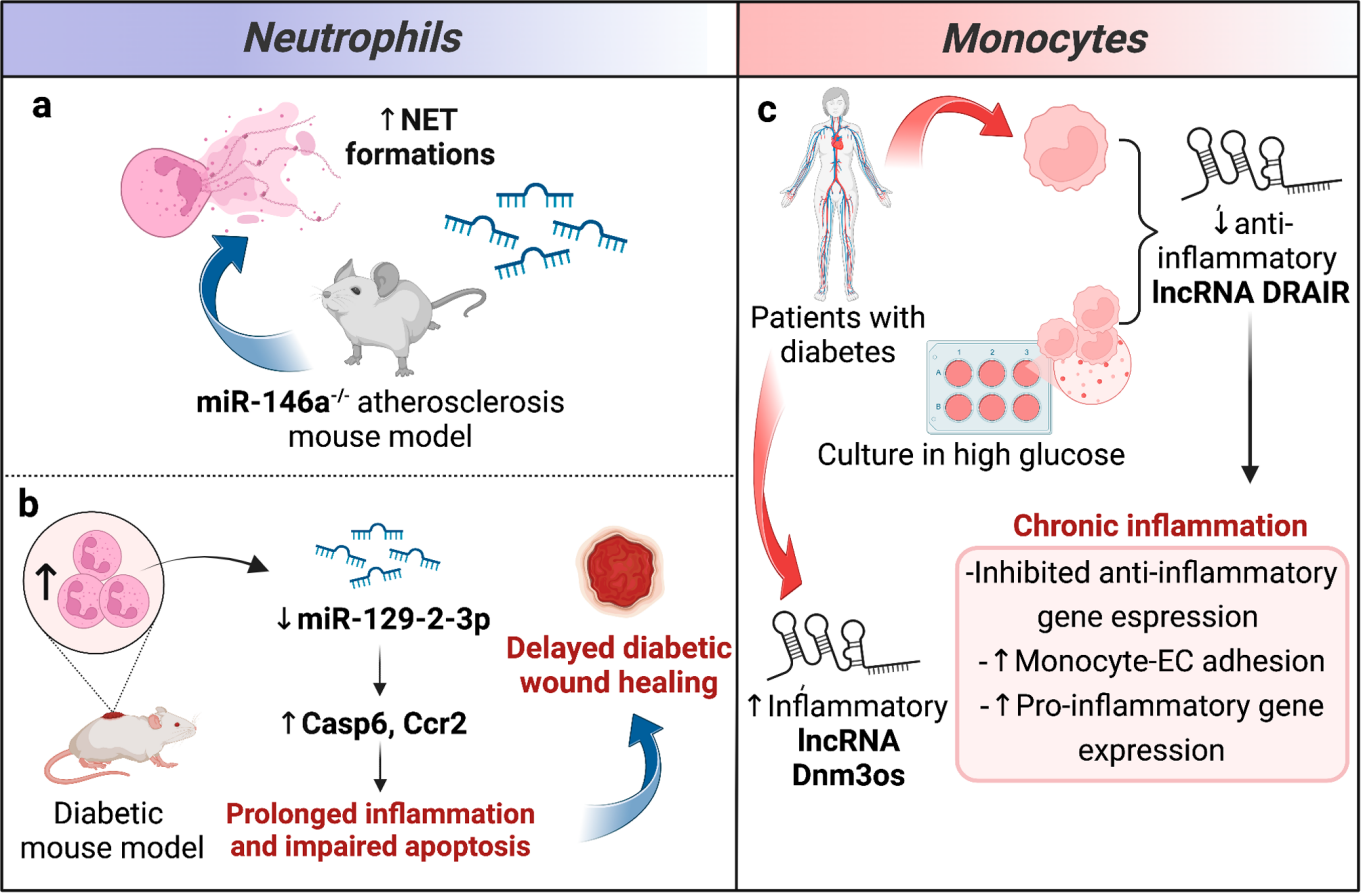
ncRNAs in the dysregulation of the innate immune system in diabetic cardiovascular complications. Examples of ncRNA-dependent deregulation in (**a, b**) neutrophils, and (**c**) monocytes in the context of DM cardiovascular complications. (**a**) miR-146 KO increases NET formation in a mouse model of atherosclerosis; (**b**) miR-129 in neutrophils mediates apoptosis and inflammation impairment in a diabetic mouse model; (**c**) Hyperglycemia leads to chronic inflammation through ncRNAs deregulation. NET: neutrophil extracellular trap, Casp6: caspase-6, CCR2: C-C chemokine receptor type 2, DRAIR: diabetes regulated anti-inflammatory RNA, Dnm3os: dynamin 3 opposite strand, EC: endothelial cell. Figure created with BioRender.com online tool

Neutrophils are continuously recruited to chronic inflammation sites in patients with DM, promoting inflammation through an excessive NET formation and impaired apoptosis which contribute to accelerated atherosclerosis and delayed wound healing respectively [82–86]. miRNAs have emerged as important regulators of inflammation-modulating neutrophils functions [87, 88]. In particular, miR146a and miR-129-2-3p are two inflammatory miRNAs in neutrophils involved in DM complications. Ana BA et al. showed that miR-146a deficiency promotes NET formations in an atherosclerosis mouse model (Fig. 2a) [89]. Previous studies demonstrated that miR-146a also has an anti-inflammatory role in monocytes, by downregulating the production of pro-inflammatory cytokines [90]. Umehara et al. observed that diabetic mice display an increase of wound neutrophils in which the downregulation of miR-129-2-3p contributes to prolonged inflammation and impaired apoptosis through upregulation of Caspase (CASP)-6 and C-C chemokine receptor type 2, thus delaying diabetic wound healing (Fig. 2b). Accordingly, overexpression of miR-129-2-3p in the skin wound site of diabetic mice enhanced the wound healing process [86]. These observations point to miRNAs as crucial elements regulating the role of neutrophils in DM complications.

### Mono/macrophages

**Fig. 3:**
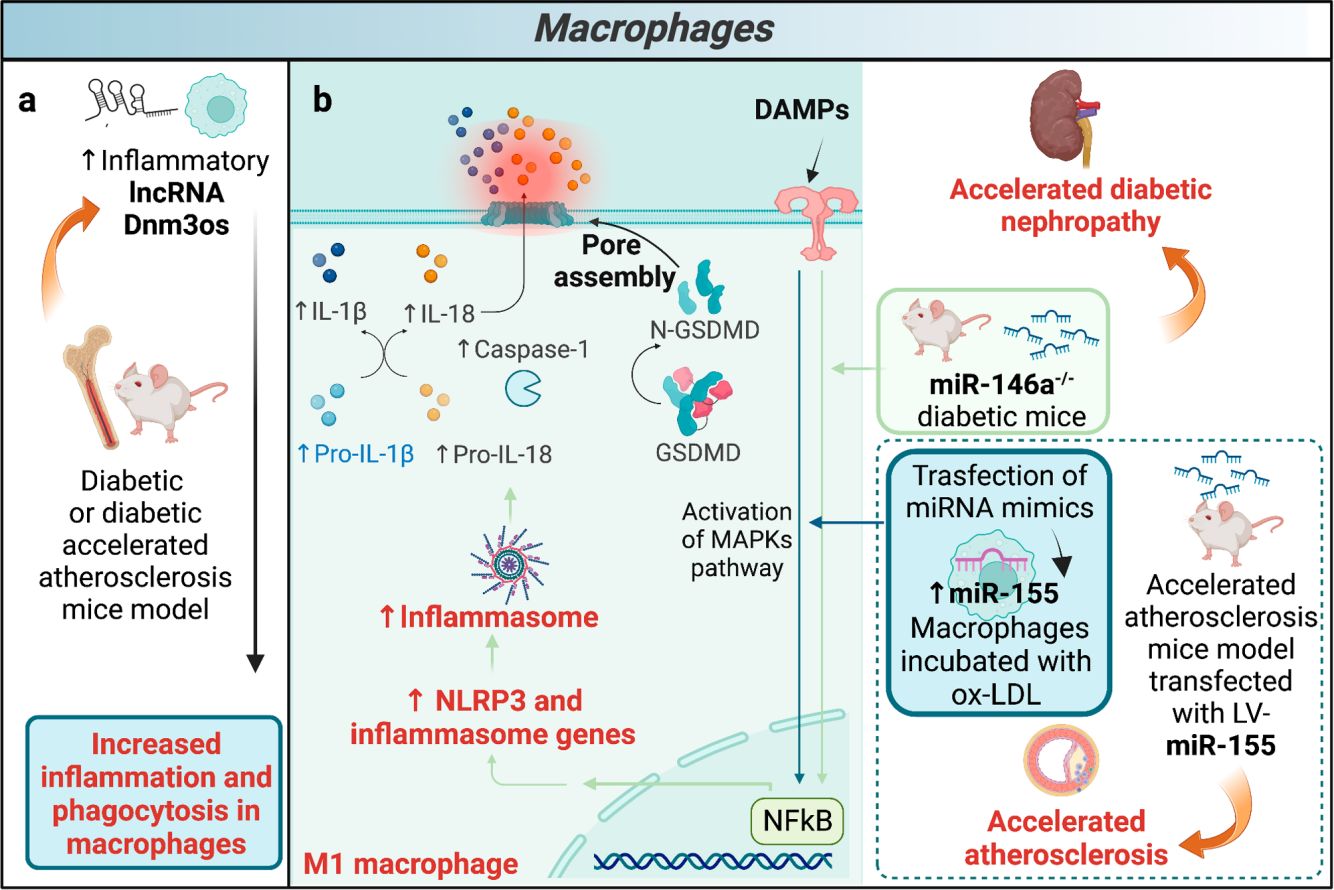
Macrophage ncRNAs in diabetes complications. (**a**) lncRNA Dnm3os, (**b**) miR-146a (green square) and miR155 (blue square) deregulations affect inflammatory signaling pathways, accelerating diabetic nephropathy or exacerbating atherosclerosis. DAMPs: damage-associated molecular patterns, IL-1β: interleukin-1 beta, IL-18: interleukin 18, GSDMD: gasdermin D, N-GSDMD: amino-terminal fragments of GSDMD, MAPKs: mitogen-activated protein kinase, NLRP3: NLR family pyrin domain containing 3, NF-κB: nuclear factor kappaB, Ox-LDL: oxidized low-density lipoprotein, AS: atherosclerosis, LV: lentiviral, Dnm3os: dynamin 3 opposite strand. Figure created with BioRender.com online tool

Like neutrophils, monocytes\macrophages are excessively recruited into chronic inflammation sites in patients with DM [91]. Two important lncRNAs, anti-inflammatory Diabetes Regulated anti-inflammatory RNA (DRAIR) and pro-inflammatory dynamin 3 opposite strand (Dnm3os) are involved in DM. DRAIR was found to be downregulated in monocytes of patients with DM compared with non-DM controls (Fig. 2c) [92]. DRAIR modulation was confirmed in vitro by culturing THP1 monocytes in normal glucose versus high glucose conditions. It was also shown that DRAIR silencing inhibited the expression of anti-inflammatory genes in THP1 monocytes. Furthermore, DRAIR silencing enhances monocyte-endothelial cell (EC) adhesion and expression of pro-inflammatory genes, such as IL-1β. Conversely, DRAIR overexpression in THP-1 cells increases monocytes/macrophage differentiation and the expression of anti-inflammatory target genes, such as cytoplasmic polyadenylation element-binding protein 2 gene and interleukin 1 receptor antagonist; at the same time, it also inhibits pro-inflammatory genes, such as tumor necrosis factor-α (TNF-α) and fc gamma receptor IIIb. Therefore, DRAIR has an anti-inflammatory role, and it has been observed that it modulates the inflammatory phenotype of monocytes *via* epigenetic mechanisms in humans. Indeed, its downregulation is associated with an inflammatory phenotype of monocytes in DM thus promoting chronic inflammation [92]. The nuclear lncRNA Dnm3os is significantly upregulated in CD14 + monocytes from patients with DM compared with healthy controls (Fig. 2c). A similar regulation of Dnm3os was observed in macrophages from high-fat diet-induced insulin-resistant mice, streptozotocin (STZ)-induced type 1 diabetic mice, and STZ-induced diabetic apolipoprotein E deficient mice (a model of accelerated atherosclerosis) compared with controls (Fig. 3a). Dnm3os overexpression increases the pro-inflammatory gene expression and phagocytosis in macrophages, while its silencing results in the opposite response. Dnm3os interacts with nucleolin which has a protective role in macrophages. Nucleolin expression levels are decreased under diabetic conditions, and this leads to Dnm3os increase, promoting inflammatory gene expression. This suggest that Dnm3os play a role in DM and accelerated atherosclerosis [93]. Emerging evidence indicates that NETs trigger NOD-, LRR- and pyrin domain-containing protein 3 (NLRP3) assembly in macrophages, thereby sustaining inflammatory response with cytokine production, e.g. interleukin (IL)-1β, IL-18 [94]. An excessive NLRP3 inflammasome activation causes tissue damage increasing the risk of developing atherosclerosis [95, 96]. Interestingly, miRNAs control the activity of NLRP3 inflammasome [97]. In particular, miR-146a and miR-155 are two of the most studied miRNAs in the field of diabetes and atherosclerosis which control NLRP3 inflammasome activity in macrophages. In intrarenal macrophages from diabetic mice, miR-146a deficiency increased NLRP3, IL-1 β, and IL-18 expression levels leading to accelerated diabetic nephropathy, ultimately confirming the protective role of miR-146a within the inflammatory signaling pathway (Fig. 3b) [98]. Also, miR-155 is involved in NLPR3 activation by regulating the mitogen-activated protein kinase kinase (MEK)/extracellular signal-regulated kinase (ERK)/nuclear factor kappa B (NFκB) pathway in oxidized LDLs (ox-LDL)-induced-macrophages. Lentivirus-mediated overexpression of miR155 in apolipoprotein E deficient mice accelerated atherosclerosis (Fig. 3b). Therefore, miR-155 may promote accelerated atherosclerosis via NLPR3 activation [99]. These two miRNAs appear to be an attractive target to attenuate diabetic inflammation by suppressing the NLRP3 inflammasome.

### Natural killer

Several ncRNAs are important regulators of NK cell functionality. In particular, two ncRNAs are involved in the NK interferon-gamma (IFN-γ) production and activation respectively. Specifically, IFN-γ antisense 1 is overexpressed in NK cells upon activation, leading to increased IFN-γ secretion [100]. Moreover, Ni et al. found that NK cells in human PB express high levels of miR-362-5p [101]. This miRNA can up-regulate the expression of CD107a (a cell surface marker exhibited by NK-activated cells), promoting perforin and granzyme B secretion. Importantly, the production of IFN-γ and the activation of NK cells have a crucial role in vascular homeostasis and the occurrence of vascular diseases [102].

### Dendritic cells

Several miRNAs, like miR-182, -148, and − 152, are known to affect dendritic cell activation. Worth mentioning, miR-181a controls the inflammatory response of DCs. Indeed, it has been observed that upon hyperlipidemia, the increase of miR-181a attenuated ox-LDL-induced DC immune inflammatory response. In addition, miR-181a inhibits the expression of an important inflammatory transcription factor, c-Fos, further corroborating miR-181a anti-inflammatory role, especially during vascular diabetic complications, like atherosclerosis [103]. Other studies confirm the importance of ncRNAs in DCs function. miR-148 and miR-152 regulate the antigen presentation of toll-like receptors (TLR) to T cells triggered by DCs [104], an important process that defends against infection, autoimmunity, and CVDs [105, 106].

### Adaptive immune cells

**Fig. 4:**
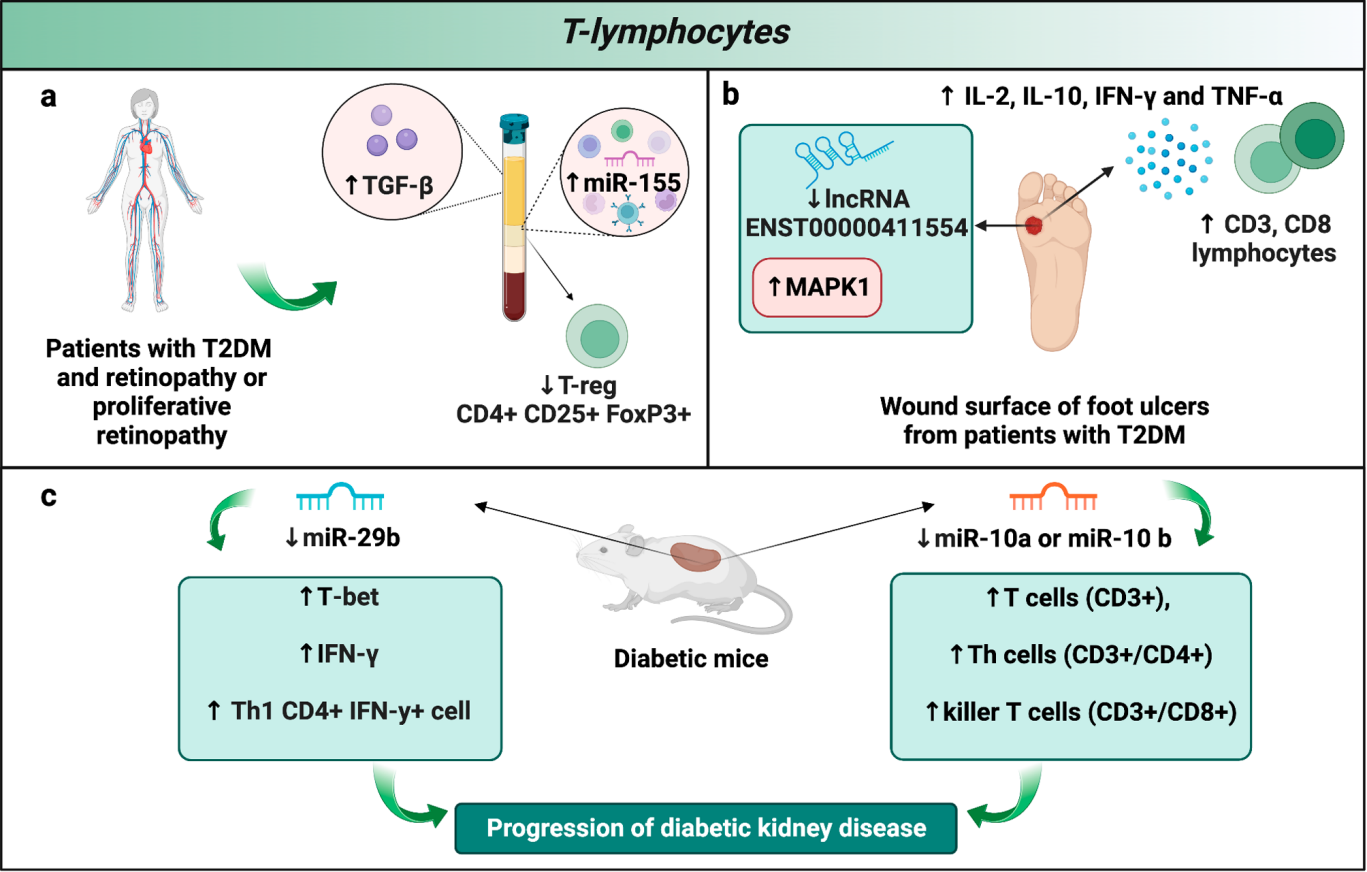
Role of ncRNAs in diabetes-related T-lymphocyte dysfunction. Examples of ncRNA-dependent T cells deregulation in (**a**) T2DM retinopathy, (**b**) T2DM wound healing and (**c**) diabetic kidney disease. T2DM: type 2 diabetes mellitus, TGF-β: transforming growth factor-beta, FoXP3: forkhead box P3, MAPK1: mitogen-activated protein kinase 1, IL: interleukin, IFN-γ: interferon-γ, TNF-α: tumor necrosis factor alpha, T-bet: T-box expressed in T cell, Th: T-helper cells. Figure created with BioRender.com online tool

T lymphocytes are major components of the adaptive immune system [107]. Several studies have shown that the immune-pathological effects mediated by ncRNAs on T lymphocytes play a role in the development of diabetic complications. In particular, four ncRNAs (miR-155, ENST00000411554, miR-29b, miR-10a, or -10b) are involved in T lymphocyte deregulation. In the PB of T2DM patients with retinopathy or proliferative retinopathy, intracellular miR-155, and serum transforming growth factor-β (TGF-β) levels are significantly upregulated while the percentage of CD4 + CD25 + forkhead box (Fox) P3 + T-regs was lower compared to non-diabetic retinopathy and healthy control group (Fig. 4a). It was shown that the expression of miR-155 was negatively related to the T-regs and TGF-β levels emphasizing that miR-155 may play an important role in the pathogenesis of T2DM retinopathy by regulating the Tregs *via* TGF-β [108]. Another study reported the altered expression of the lncRNA ENST00000411554 in the wound surface of foot ulcers from patients with T2DM and found an association with T lymphocytes impaired functions and immune regulation imbalance (Fig. 4b). In particular, Xu et al. observed that the diabetic wound was infiltrated with higher levels of CD3, CD8 lymphocytes, and inflammatory factors, i.e. IL-2, IL-10, IFN-γ, and TNF-α, compared to non-diabetic control. They performed a microarray on the tissue of diabetic foot ulcers to investigate the molecular mechanisms involved in immune dysfunction. Analyses showed the downregulation of 13 lncRNAs, and, among them, of lncRNA ENST00000411554, which is located in a key upstream regulatory region of the target gene Mitogen-Activated Protein Kinase (MAPK) 1. Interestingly, an inverse correlation between the lncRNA and MAPK1 was observed. Therefore, it was hypothesized that ENST00000411554 could be involved in immune-regulation imbalance of T2DM, thus regulating the expression of inflammatory factors in T-cell-induced responses by downregulating MAPK1 [109]. Several studies indicate miRNAs as regulators of T lymphocytes activities in diabetic nephropathy. As an example, renal miR-29b downregulation contributed to the upregulation of T-Box Expressed in T cells and IFN-γ at both mRNA and protein levels, promoting CD4 + IFN-γ + Th1 cell infiltration in db/db mice (Fig. 4c). Overexpression of miR-29b inhibited Th1-associated immune injury [110]. Similarly, miR-10a or -10b downregulation promoted the infiltration of T cells (CD3+), Th cells (CD3+/CD4+), and killer T cells (CD3+/CD8+) in the kidney from STZ-treated diabetic mice (Fig. 4c) [111]. Although several studies have investigated the role of B lymphocytes, more investigations are needed to understand the role of B-lymphocyte ncRNAs in this context.

## Extracellular vesicles shuttle ncRNAs between immune and cardiovascular cells

### Cell-to-cell communication mediated by extracellular vesicles

**Fig. 5:**
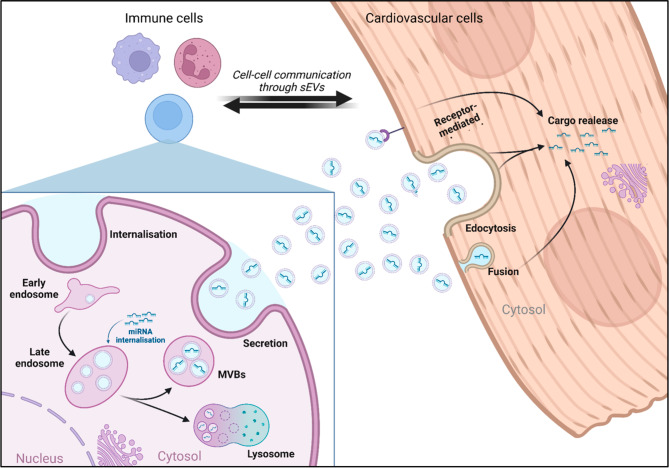
ncRNA transfer between immune and cardiovascular cells. Early endosome in immune cells matures into late endosome, internalizing ncRNAs in the process. Depending on their pH and molecular content, late endosomes can further mature either into lysosomes or into multivesicular bodies. Multivesicular bodies then fuse with the plasma membrane releasing their EVs content in the extracellular milieu. EVs are subsequently internalized by target cardiovascular cells through either receptor-mediated endocytosis or membrane fusion, releasing their cargo in the cytoplasm of target cells. Cardiovascular cells can in turn signal back to target immune cells releasing their EVs. EV: extracellular vesicle, MVB: multivescicular body, sEV: small extracellular vesicle. Figure created with BioRender.com online tool

The cells in the adult human heart and vasculature are continuously engaged in cell-to-cell communications (CCC) to regulate both single-cell functions and coordinated responses needed for homeostasis [112]. CCC is ensured by a variety of biochemical mediators and electrical impulses [113, 114]. Diseases disrupt CCC, and cells might not interact properly or decode molecular messages properly. The study of altered cellular crosstalk might facilitate an understanding of disease pathogenesis and progression. Although protein-mediated interactions, such as ligand-receptor, receptor–receptor, and receptor-extracellular matrix interactions, have been better studied so far [114, 115], CCC can also take place via ncRNAs, and miRNAs in particular. miRNA-based CCC takes advantage of the interaction between miRNAs released by “message sender cells” and the miRNAs “target genes” present in the “message recipient cells”. miRNA exchanges are mostly realized *via* extracellular vesicles (EVs) (Fig. 5), although several alternative extracellular carriers of miRNAs, such as lipoproteins and argonaute-2, have been described [115]. Moreover, miRNAs can also be exchanged via connexin gap junctions, without being released extracellularly.

The term EVs denotes a highly diverse family of membrane vesicles of different biogenesis and sizes: apoptotic bodies (> 1000 nm), microvesicles (MVs) (150 nm − 1 μm), and small EVs (sEVs, circa 30–150 nm). sEVs, also called “exosomes”, are currently of particular interest to the biomedical community. sEVs are actively secreted and contain endosomal membrane markers, such as tetraspanins, plus a composite molecular cargo; that can include RNAs and proteins [116–118], as well as mitochondria fragments and cytosol-derived small molecules (< 1500 Da), including metabolites as sugars, amino acids, lipids, nucleotides, and N-glycans [119, 120]. Once released by their parent cells, sEVs protect their molecular cargo from degradation until they pass it in a functionally active status to neighboring cells through binding, fusion, or endocytosis [117]. Alternatively, sEVs are transported in biological fluids to reach distant organs [121] or be excreted [118]. sEVs shuttle biologically active miRNAs from their parent cell to recipient cells, thus spreading the miRNA regulatory actions and profoundly influencing biological functions, including pro and anti-angiogenic responses [122–124].

The adult human heart comprises billions of cells and approximately a third of them are cardiomyocytes, whilst the remainder comprises ECs and other vascular cell types, fibroblasts, resident macrophages, and neural cells [125, 126]. Recent evidence shows that different types of cardiovascular cells use sEVs as a means to communicate with each other as well as with other cells present in the cardiac microenvironment, such as immune cells and adipocytes [122, 124, 127, 128]. Moreover, evidence is accumulating for different types of sEVs being simultaneously released by a single cell type [129]. sEVs can differ substantially in their cargo and membrane composition [130, 131]. Consequently, the pool of sEVs that populate the heart’s extracellular space and contribute to cell-to-cell communications is diverse and variable. The coordination of sEV-mediated responses is important for maintaining physiology, but it can also mutate to support disease propagation [132, 133]. This can be understood considering that the amount and quality of sEVs secreted from a cell are influenced by both its activation status and the external environment to which the cell is exposed [134, 135], including hypoxia and D-glucose concentration [136]. As an example, Wang et al. showed that rat diabetic cardiomyocytes produce anti-angiogenic sEVs *in vitro*. This is in net contrast with the proangiogenic program activated in recipient ECs by their non-diabetic sEV counterparts [124]. Also, macrophages and ECs communicate *via* EVs, and changes in the macrophage phenotype contribute to reduced microvascular repair and impaired myocardial perfusion by promoting atherosclerosis [132], chronic inflammation and fibrosis [130, 137, 138].

### Extracellular vesicles mediated transfer of ncRNAs in diabetes

The chronic inflammation induced by DM dysregulates the constant interaction of immune cells and cardiovascular cells [57]. CCC in DM has been reported to be driven mainly by sEVs RNA cargo [139–142]. Indeed, higher levels of EVs have been found in human and animal models of type 1 and 2 DM [141]. miRNAs are the most studied ncRNA cargo of sEVs with a demonstrated functional role. sEVs-miRNAs have been shown to act on pancreatic β-cells, the insulin-producing cells. In detail, Zhang et al. showed that miR-223-3p and − 5p were two to three times increased in the medium of cultured pancreatic β-cells after high glucose stimulation, which modulated Glucose transporter 4, favoring insulin sensitivity [142]. Of note, several studies revealed bidirectional crosstalk between β-cells and immune cells via EVs, both in physiological and pathological conditions [143–146]. As such, metabolic regulatory miRNAs, such as miR-29a-3p [147], miR-155-5p [148], miR-503 [149], and miR-210-3p and − 5p [150], can be carried by EVs and delivered into insulin-responsive cells and organs via paracrine or endocrine routes.

Macrophage-derived sEVs also play a role in diabetic complications. It has been reported that high glucose levels in pro-inflammatory macrophages induce the production of EVs carrying high levels of IL-1β, inducible nitric oxide synthase, human antigen R (HuR), miR-21-5p, miR-486-5p, and TGF-β mRNA [151–155]. These biomolecules can be transferred to target cells and subsequently induce renal and cardiac injury and dysfunction. In particular, two miRNAs closely related to cardiac fibrosis and diastolic dysfunction, miR-122-5p [156–158] and miR-21-5p are sorted specifically into EVs by the retinol-binding protein HuR [159]. Additionally, sEVs can effectively transmit pathogenic miRNAs to target cells, including vascular smooth muscle cells (VSMCs), ECs, neutrophils, and macrophages, leading to vascular stenosis, dysfunction, and inflammation, promoting atherosclerosis and thrombosis [157]. Likewise, recent mouse studies suggest that T cells might also have a role in DM by communicating with β-cells through the transfer of pro-apoptotic sEVs-miR-142-3p/5p and miR-155 [158]. As mentioned above, CD34 + HSPCs can transfer a negative pro-apoptotic signal including ncRNAs to the ECs *via* sEVs [14].

Exposure to numerous noxious stimuli, such as other cardiovascular risk factors, is also considered an important pathogenic event in DM [160]. ECs activation entails the expression of cell adhesion molecules like intercellular adhesion molecule 1 (ICAM-1) and vascular cell adhesion molecule 1 (VCAM-1) that serve as triggering signalling to the leukocyte adhesion on the endothelium. The uptake of LDLs into the intima and its oxidation, with the enhanced infiltration of monocytes/macrophages, promotes the formation of foam cells that induce cytokine and chemokine production and the additional recruitment of circulating immune cells [61]. As an example, miR-205-3p/-5p can regulate the adhesive properties of ECs, most likely by targeting the tissue metallopeptidase inhibitor 3, thereby triggering the release of soluble TNF-α, increasing monocyte adhesion [161]. Similarly, numerous miRNAs (miR-181b-5p, miR-103, miR-126-3p, miR-10a-5p, miR-92a-3p, let-7 g-3p/-5p, miR-195-5p) regulate expression in ECs of cytokines with pro or anti-inflammatory potential by indirectly targeting NF-kB and consequently affecting the immunophenotyping [162–164].

In addition to miRNAs, lncRNAs, and circRNAs can also be exchanged between cells *via* EVs, and play a regulatory role in DM complications. For example, lncRNA Nexilin F-actin binding protein antisense RNA 1 (NEXN-AS1) circulates in sEV and suppresses the TLR4/NF-kB pathway, reducing endothelial activation and monocyte recruitment in human vascular ECs [165]. Further, its overexpression inhibits pro-inflammatory pyroptosis-related biomarkers known to drive atherosclerosis, such as NLRP3, CASP-1, IL-1β, IL-18, Gasdermin D [166]. Moreover, lncRNA growth arrest specific 5 (GAS5), long intergenic non-protein coding RNA 1005 (LINC01005), and metastasis associated lung adenocarcioma transcript-1 (MALAT-1) have also been detected in sEVs and may play a role in DM pathogenesis [167–169]. In detail, silencing lncRNA GAS5 alleviates apoptosis and fibrosis in diabetic cardiomyopathy by targeting miR-26a/b-5p [170]. Besides, sEVs-LINC01005 from ECs has been reported to promote VSMC phenotype switch, proliferation, and migration by regulating the miR-128-3p/Kruppel-like factor (KLF) 4 axis [168]. On the contrary, sEVs-MALAT-1 derived from ECs promoted anti-inflammatory macrophage polarization [169, 171].

Of relevance, the circRNA homeodomain-interacting protein kinase 3 (circHIPK3) released by ECs-sEVs has been reported to promote the proliferation of VSMCs induced by high glucose via the miR-106a-5p/FoxO1/VCAM1 pathway [172]. Moreover, sEVs- circRNA euchromatic histone-lysine-N-methyltransferases 1 from pericytes could suppress the generation of NLRP3 inflammasomes in high glucose-treated ECs by upregulating the levels of the transcription factor nuclear factor I A, which in turn inhibited the apoptosis of ECs and reduced its angiogenic activity, ultimately attenuating high glucose-induced retinal vascular dysfunction [173].

## Effect of aging and sex on diabetes-related CVDs: the role of ncRNAs

A large body of evidence shows sex-based differences in the pathophysiology of CVDs and the response to cardiovascular treatments [174–181], which also implies underlying ncRNA-mediated regulatory mechanisms in a sex-dependent manner [182]. In this context, there are pronounced sex differences in the impairment of glucose metabolism, with men presenting more frequently elevated fasting glucose levels, while women more frequently develop impaired glucose tolerance [183–186]. In addition, the menopause transition accelerates the risk for CVDs [187, 188]. The influence of estrogen decline with menopause on risk factor clustering increases, thereby leading to hormonal regulation of body fat distribution and related metabolic abnormalities [189, 190]. In turn, together with aging, these elements can account for CVDs in postmenopausal women [191, 192]. Along this line, altered glucose homeostasis leading to metabolic changes [193] may predispose postmenopausal women to the development of T2DM [194]. In this regard, in human ECs estradiol modulates regulatory networks composed of miRNAs, transcription factors, and downstream genes.

### miRNAs associated with diabetes mellitus, aging, and sex

Interestingly, aging and DM share common pro-inflammatory pathways, such as the NF-κB and NLRP3 signaling pathways, which are regulated by several miRNAs. Among the most remarkable miRNAs regulating age-mediated inflammation, miR-146a stands out, as it can reduce NF-κB DNA binding activity [195] and has been proposed as a biomarker of healthy aging [196]. Inflammation is a common ground in several CVDs and sex differences in the transcriptomic regulation of inflammatory genes and pathways in CVDs have been previously reported [181, 197, 198]. Circulating levels of miR-146a decline in aged people, and this phenomenon is particularly evident in men [196]. In turn, miR-146a levels decrease after estrogen treatment in mouse-isolated splenic lymphocytes [199], and it could act as a sex-biased inflammation-associated biomarker, modulating T2DM and other aging-associated diseases [200]. Also, miR-34a, a key promoter of vascular aging related to DM-associated vascular complications, shows sex differences in its expression [201]. Of note, it induces a systemic inflammatory state [202]. Indeed, miR-34a expression is elevated in several cardiac pathologies, and its therapeutic inhibition in a mouse model of dilated cardiomyopathy provides more protection in females than in males [203]. Particularly, in association with DM, sex was found to be a determinant of plasma miR-34a levels in patients with left ventricular diastolic dysfunction [204].

Other miRNAs, such as let-7 g and miR-221 have been related to glucose metabolism [205–207]. Thus, different studies support a sex-dependent regulation of physiological functions by ncRNAs in proinflammatory states that underlie cardiovascular or metabolic diseases, among others, also show sex differences, as women with metabolic syndrome display higher circulating levels of let-7 g and miR-221 than men [207]. In addition, mouse studies have revealed a male-specific induction of miR-23a-3p, miR-27b-3p, miR-130a-3p, miR-133a-3p, miR-143-3p, and let-7e-5p, together with a corresponding downregulation of downstream protein targets involved in mitochondrial metabolism, thereby probably contributing to sex-biased maladaptive cardiac remodeling [208].

### Intrauterine/placental RNA modulation and gestational DM

Some of the sex differences in transcriptomics are evident from fetal age. In particular, analysis of miRNA expression patterns in healthy placentas performed in the Environmental Influences on Early Ageing (ENVIRONAGE) study shows a different miRNA expression pattern in the placenta of mothers of girls and boys, and enrichment analyses showed sex-dependent differences in the regulation of biological processes, including metabolic and immunological pathways [209]. In newborn girls, the higher expression of placental miRNA-210, miR-146a, miR-34a, and miR-222, among others, is critical for specific cellular processes in aging and inflammation [210]. It is important to note that some of the alterations that occur during pregnancy can affect the future development of pathologies in both the mother and the newborn [211]. In particular, women with gestational DM have a higher risk of developing future T2DM [212]. In this regard, during gestational DM, the miRNA expression profile changes in human placental ECs [213]. Thus, the hyperglycemic intrauterine environment could affect placental function through elevated levels of proinflammatory cytokines, as well as through transcriptional modifications, including those mediated by ncRNAs.

## Circulating immune-associated ncRNAs as biomarkers of diabetic CVDs

Extracellular ncRNA levels are dynamically regulated in response to stress and diseases providing an opportunity for the identification of biological markers [214]. Indeed, circulating ncRNA biomarkers may reflect the physiological or pathological status of a subject. The analysis of ncRNA patterns may lead to the development of non-invasive tools for clinical decision-making in the fields of diagnosis, prognosis, and therapeutic guidance [215, 216].

Several studies have already provided relevant data on the potential use of immune-associated ncRNAs in the context of the cardiovascular complications of DM, the miRNAs being the main subclass investigated (Table 3). For instance, Zampetaki et al. [217] provided indirect evidence on the role of these transcripts in the link between the pathophysiology of DM and the manifestation of its vascular complications. The authors demonstrated the alteration in the plasma levels of miRNAs implicated in vascular diseases, e.g., miR-126, in prevalent DM. Supporting these results, Jansen et al. [218] reported the deregulated levels of miR-126 in circulating MVs from patients with DM and that patients with reduced miR-126 levels are at higher risk for the occurrence of concomitant coronary CAD. Another remarkable example of immune-associated miRNAs as indicators of vascular complications is given by miR-15a and miR-16a. The serum levels of both miRNAs are upregulated in patients with CLI and positively correlated with amputation after restenosis at 12 months post-revascularization in T2DM patients with CLI [78].

**Table 3:** Examples of the potential role of ncRNAs as biomarkers of cardiovascular complications of diabetes

miRNAs			
Reference	Study Population	Source	Main findings
[78]	122 patients with CLI and T2DM, 20 patients with CLI & 43 non-ischemic and non-diabetic subjects	Serum	miR-15a and miR-16 positively correlate with the risk of amputation after restenosis
			Serum miR-15a is positively associated with post-revascularization restenosis considered as the first event
[221]	90 non-HF, 90 HFpEF & 90 HFrEF	Serum	Reduced levels of miR-146a-5p in HF patients compared with the control group
			Combination with contemporaneous laboratory parameters provides an optimal discriminative value
[218]	55 patients with DM & 80 non-DM patients	Circulating microparticles	miR-26a and miR-126 are significantly reduced in DM patients compared to controls
			Patients with reduced miR-26a and miR-126 expression levels are at higher risk for the occurrence of a concomitant CAD
[219]	86 patients with well-controlled T2DM	Serum	miR-1 and miR-133a levels are independent predictors of myocardial steatosis
[222]	28 healthy controls, 26 patients with DM, 22 patients with chronic HF & 15 patients with both DM and chronic HF	Plasma	miR-30c is reduced in patients with DM and HF
			miR-30c levels are negatively correlated with plasma glucose levels in patients with chronic HF
[223]	63 patients with DM with and without cardiac dysfunction	Plasma	miR-144 is significantly decreased in the plasma of patients with DM with cardiac dysfunction
			Plasma miR-144 could serve as a specific predictor of patients with DM developing cardiac dysfunction
**Long non-coding RNAs**			
[232]	414 patients with acute MI	Whole Blood	MIAT is a predictor of LV dysfunction at 4 months after MI
	56 patients with diabetic cardiomyopathy, 44 patients with DM but without cardiomyopathy & 42 healthy controls	Serum	HOTAIR is a diagnostic biomarker of diabetic cardiomyopathy
[234]	6 patients with DMs and 6 healthy subjects	Serum	KCNQ1OT1 is elevated in patients with DM
**Circular RNAs**			
[234]	45 patients with T2DM and 45 healthy subjects	Peripheral white blood cells	circANKRD36 is associated with chronic inflammation in T2DM patients

DM: diabetes mellitus, CAD: coronary artery disease, circANKRD36: circular ankyrin repeat domain 36, CLI: critical limb ischemia, HF: heart failure, HFpEF: heart failure with preserved ejection fraction, HFrEF: heart failure with reduced ejection fraction, HOTAIR: homeobox transcript antisense RNA, KCNQ1OT1: potassium voltage-gated channel subfamily Q member 1 opposite strand/antisense transcript 1, LV: left ventricle, MI: myocardial infarction, MIAT: myocardial infarction-associated transcript, T2: type 2.

Concerning diabetic cardiomyopathy, serum miR-1, and miR-133a are independent predictors of myocardial steatosis in patients with well-controlled T2DM patients [219]. Both miRNAs may be useful biomarkers for the evaluation of myocardial steatosis in T2DM, an early manifestation in the pathogenesis of cardiac-related complications which quantification is currently impractical for large-scale population screening. In line with these findings, miR-146a-5p is a key regulator of the immune response, inflammation, and fibrosis in diabetic cardiomyopathy [220] and its circulating levels have been proposed as a biomarker of HF [221]. The plasma levels of miR-30c are reduced in patients with DM and HF and its levels are negatively correlated with plasma glucose concentration in chronic HF [222]. Additionally, Tao et al. [223] reported that plasma miR-144 is downregulated in the plasma of patients with DM with cardiac dysfunction, compared to patients with DM without cardiac dysfunction, and proposed this miRNA as a predictor of cardiac dysfunction in DM patients. Also, given the involvement of KLF5 in diabetic cardiomyopathy, it is also relevant that, miR-145, and miR-375 target KLF5, thereby repressing its levels [224–226]. While the role of these miRNAs as potential circulatory biomarkers is yet to be explored, there are indications of the mechanistic involvement of at least miR-145 in the pathological process contributing to the development of diabetic cardiomyopathy [227–229].

More recently, other ncRNA classes, such as lncRNAs, have been proposed as tools for disease management [230], including immune-associated lncRNAs as indicators of DM-induced CVDs. The long-non-coding myocardial infarction–associated transcript (MIAT), which is upregulated in serum and PB-mononuclear cells from T2DM patients [231] and predominantly expressed in lymphocytes, has been described as a predictor of left ventricle dysfunction at 4-month follow-up in patients that suffered an acute MI [232]. The expression of the lncRNA Homeobox transcript antisense RNA (HOTAIR) is downregulated in myocardial tissues and the serum of patients with diabetic cardiomyopathy compared with both patients with DM but without cardiomyopathy and healthy controls. Interestingly, the serum levels of this lncRNA discriminate between patients with diabetic cardiomyopathy and healthy controls [233]. In addition, the lncRNA Potassium Voltage-Gated Channel Subfamily Q Member 1 Opposite Strand/Antisense Transcript 1 (KCNQ1OT1), associated with pyroptosis and fibrosis in diabetic cardiomyopathy, is significantly elevated in the serum of patients with DM compared with healthy subjects [234].

Advances in RNA sequencing have also drawn attention to circRNAs. The evidence on immune-associated circRNAs as biomarkers of the cardiovascular complications induced by DM is still scarce. Nevertheless, some data point to a role as an indicator of pathological mechanisms linked to these conditions. Fang et al. [235] identified circular ankyrin repeat domain 36 (circANKRD36) as a mediator of inflammation-associated pathways in T2DM patients. Interestingly, circANKRD36 was upregulated in PB leucocytes and correlated with inflammatory cytokines involved in CVDs such as IL-6 and TNF-α.

Therefore, immune-associated ncRNAs emerge as an interesting tool to evaluate the cardiovascular complications associated with DM. The translation of these transcripts to clinical practice may improve patient management and decision-making. Of note, circulating ncRNA quantification does not require any special infrastructure, since they can be quantified in clinical specimens (plasma and serum) with techniques (quantitative PCR) currently used in clinical laboratories. Nevertheless, their incorporation into clinical laboratories needs to overcome several challenges, including the improvement of reproducibility, which is beyond the scope of the current article and discussed here.

## ncRNAs as therapeutic targets in diabetic cardiovascular complications

### Strategies to modulate ncRNAs-ncRNA therapeutics: experiences in diabetic CVDs

The epidemic of diabetic cardiovascular complications underlines the need for the translation of current preclinical knowledge and novel technologies into actual clinical practice. Recently, ncRNAs were shown to be powerful therapeutic tools for different disease settings by affecting the onset and progression of several human disorders [236]. Therefore, researchers focused on the development of innovative next-generation technologies to exploit these ncRNAs to treat such diseases, especially those not susceptible to pharmaceutical interventions [237].

**Fig. 6:**
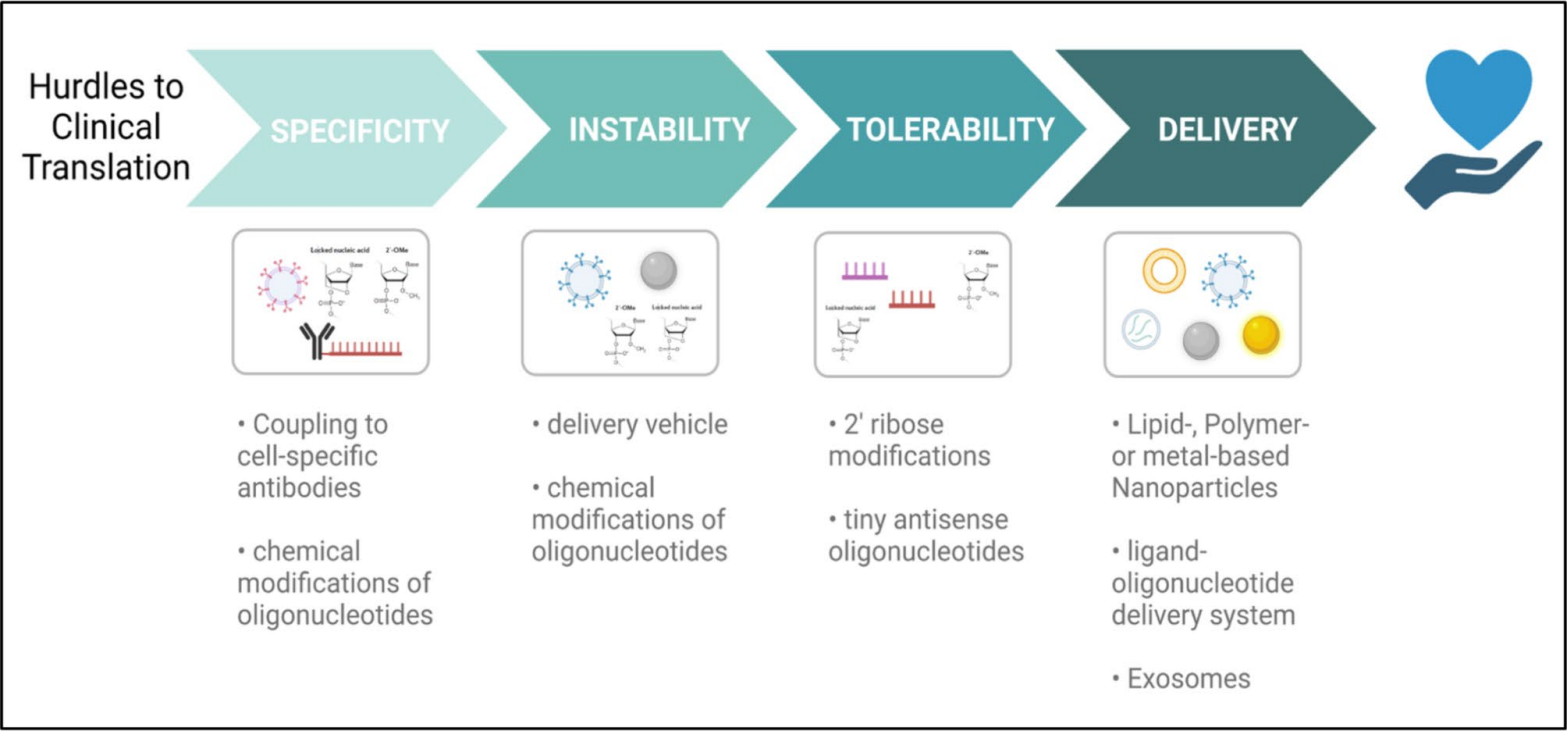
Key points for the delivery of ncRNA-based therapies. An ideal ncRNA-based therapy is: specific, as it targets a specific cell type of interest; stable as it needs to reduce intrinsic ncRNA instability; tolerable, as it must not trigger the host immune response; deliverable, as it must include a suitable delivery vehicle. Figure created with BioRender.com online tool

The use of ncRNA-based therapies has several advantages compared to other compounds. NcRNAs, and in particular miRNAs, can naturally modulate different specific targets within one pathway thereby exerting a broader biological effect. Thus, ncRNA therapies might have the potential to boost therapeutic effects compared to those drugs acting on one single target gene. Additionally, chemical modifications can easily be adapted to prolong the stability and half-life of ncRNA drugs in circulation, facilitating the effective delivery to the site of interest without degradation (Fig. 6).

Strategies to target the level of ncRNAs can be classified into two approaches: suppressing upregulated ncRNAs or enhancing levels of downregulated ncRNAs. Technologies to modulate miRNA expression include miRNA mimics to elevate their expression; concerning miRNA inhibition, miRNA sponges exert their function by sequestration of endogenous miRNAs, while inhibitory chemical compounds can compromise miRNA action through the interaction with components of miRNA biogenesis pathways. Moreover, the most pursued strategy relies on base pairing of complementary antisense oligonucleotides (ASOs) with ncRNA sequences leading to duplex formation or subsequent degradation (e.g. anti-miRNAs). LncRNAs can be targeted through modified ASOs such as locked nucleic acids (LNAs) with modified phosphorothioate backbones, gapmers composed of two LNA flanking regions and a central DNA stretch that activates ribonuclease H cleavage, or small interfering RNAs inducing their degradation [237, 238]. Other modulation tools include genome-editing strategies such as Clustered Regularly Interspaced Short Palindromic Repeats (CRISP)/CRISPR associated protein 9 (Cas9) or viral-based approaches [239, 240]. Although there is an increasing number of preclinical reports regarding ncRNA therapies, only a small amount have proceeded into clinical trials [241, 242]. Key challenges have to be faced to successfully translate ncRNA therapeutics into the clinic. Off-target effects result in suppressing genes other than the desired target due to sequence similarities or overdosing along with undesired immune responses. Additionally, special delivery systems are required to target the organ and cell type of interest and naked oligonucleotides have to be chemically modified to resolve issues such as degradation by nucleases or their inefficient uptake due to negative charge or size [243–245]. In this respect, recently, it has been shown that lipid- or polymer-based nanoparticles, as well as exosomes, are promising delivery tools (Fig. 6) [246–250].

Nonetheless, a plethora of RNA-based drugs are already under clinical development, most of them aiming to modify mRNA levels in the liver, central nervous system, or muscle and some of them have even been approved by the Food and Drug Administration or European Medicines Agency (e.g. Viltolarsen, Nusinersen, Patisiran) [251–254]. In addition, tremendous progress has also been made in the field of miRNA-based drugs which entered clinical testing [238, 255] and lncRNA-based formulations are also gaining more interest as promising treatment options. One of the ncRNA compounds, which entered the clinical phase, is an inhibitor against miR-92a (MRG-110) [256, 257] that was shown to exhibit beneficial effects in wound healing and CVDs such as LI, MI (NCT03603431, NCT03494712). Another example is anti-miR-21, which was evaluated for its capability to reduce kidney fibrosis in patients with Alport syndrome (NCT03373786) and will be further examined in a clinical phase II study (NCT02855268) [258]. Moreover, CDR132L, a synthetic oligonucleotide inhibiting miR-132 indicated as a key regulator in cardiac remodeling processes [259, 260], entered recent clinical study phase II (NCT04045405, NCT05350969) for safety and efficacy assessment in ischemic HF patients. Concerning DM, AstraZeneca performed a phase I/IIa clinical trial (NCT02612662, NCT02826525) with an N-Acetylgalactosamine (GalNAc)-conjugated anti-miRNA against miR-103/107 (RG-125) [261]. miR-103/107 is an insulin sensitizer already tested in preclinical models that show promise as a compound to treat patients with T2DM and nonalcoholic fatty liver diseases.

### Anti-diabetic and anti-inflammatory drugs affecting ncRNAs

#### Antidiabetic drugs and ncRNAs

Antidiabetic drugs can impact the cellular epigenetic landscape, providing possible interactors of these drugs. In this respect, metformin is one of the most studied drugs. Metformin is a biguanide derivative and a mainstay prescribed drug for the treatment of T2DM [262]. Metformin’s primary effect is mediated by the inhibition of hepatic gluconeogenesis, but it can also ameliorate the “inflammaging” process, increase vascular function, and improve lipid profiles affecting the outcomes of CVDs [263, 264]. Interestingly, metformin can reduce ROS generation and contrast oxidative stress-induced apoptosis and inflammation in cardiomyocytes and ECs via AMP-activated protein kinase activation [265]. In particular, metformin treatment increased the levels of miR-20a-5p, miR-34a-5p, miR-130a-3p, miR-106b-5p, miR-125b-3p, and let-7c [266], and of the miR-200s family (miR-200a-3p, miR-141-3p, and miR-429), previously associated to senescence and aging (Table 4) [267, 268]. Moreover, Giuliani et al. reported that metformin can revert the senescence-associated modulation of miR-216-3p, -216-5p, and − 217-5p, which were previously identified among the most upregulated miRNAs in senescent ECs [269].

**Table 4:** Anti-diabetic, anti-inflammatory/anti-aggregation drugs impacting on ncRNAs

Antidiabetic drugs and ncRNAs			
Reference	Study Population/experimental system	Treatment	Main findings
[333]	Mouse liver	Mice treated with metformin	Metformin induced the expression of miR-20a-5p, miR-34a-5p, miR-130a-3p, miR-106b-5p, miR-125b-3p, and let-7c
[334]	BJ-1s human neonatal foreskin fibroblasts	Cells treated with metformin at different passages	Metformin induced the expression of miR-200a, miR-141, miR-429 and of miR-205 in senescent BJ-1s cells
[335]	Human umbilical vein ECs	Cells treated with metformin at different passages	Metformin induced the expression of miR-100-5p, miR-125b-5p, miR-654-3p, miR-217 and miR-216a-3p/5p in senescent ECs
[270]	Mouse bone marrow-derived endothelial precursor cells	Cells treated with palmitic acid and metformin	Metformin reverted angiogenesis impairment caused by palmitic acid by attenuating miR-130a/p-AKT axis and increasing PTEN expression
[336]	Mouse microvascular ECs	Cells exposed to HG and treated with metformin	Metformin reduced and increased miR-34a-5p and SIRT1 expression levels, respectively, attenuating HG-induced angiogenesis impairment
[337]	Neonatal rat ventricular cells	Cells exposed to H_2_O_2_ and treated with metformin	Metformin reverted H_2_O_2_- and ischemia/reperfusion-induced miR-1a-3p expression, reducing cell death
	Mouse ischemia/reperfusion	Hearts of mice that underwent the I/R injury and treated with metformin	
[273]	Mouse ischemia/reperfusion	Hearts of mice that underwent the I/R injury and treated with metformin	Metformin reduced I/R induced-miR-34a-5p expression
	H9C2 rat cardiomyocyte cells	Oxygen-glucose deprivation/recovery and treatment with metformin	Metformin reduced miR-34a-5p levels through decreasing SIRT1-p53 activity
	90 ACS (STEMI) patients	Metformin 3-months treatment pre-infarction	Metformin reduced serum miR-34a levels and CKMB activity and mitigated PCI-induced reperfusion injury
	Thoracic aortas of diabetic rats	Liraglutide treatment	Liraglutide reduced miR-34a-5p and increased the anti-apoptotic protein Bcl2 and SIRT1, contrasting cell death
[280]	25 patients with T2DM	Serum from patients with DM treated with liraglutide	Liraglutide induced the expression of miR-130a-3p, miR-27b-3p, and miR-210-3p
[338]	Mouse MCAO	Brain after MCAO and metformin treatment	Metformin reduced H19-induced oxidative stress injury
[282]	10 frail old adults with HFpEF and DM	Whole blood after 3 months-treatment with empagliflozin or metformin or insulin	Empagliflozin specifically reduced miR-21 and miR-92 levels compared to metformin- or insulin-treated HFpEF patients and to controls
**Anti-inflammatory/anti-aggregation drugs and non-coding RNAs.**			
**Reference**	**Study Population/experimental system**	**Treatment**	**Main findings**
[339]	46 ischemic stroke patients	PBMNCs from stroke patients treated with ASA for 10 days	ASA induced miR-145-5p levels in stroke patients
	VSMCs	Cells treated with ASA	ASA increased miR-145-5p and decreased CD40 levels, respectively, reducing VSMCs proliferation
[340]	Platelets of 12 ASA -treated CVD patients (6 with low and 6 high platelet reactivity)	ASA treatment	miR-135a-5p and miR-204-5p levels correlated with platelet reactivity
[341]	Platelets of 945 acute coronary syndrome patients	ASA treatment	-Lower miR-19b‐1‐5p expression was associated to ASA insensitivity and to a higher risk of MACCE - Low miR-223 was a predictor of responsiveness to antiplatelet therapies

ACS: acute coronary syndrome, ASA: acetylsalicylic acid, Bcl2: B-cell lymphoma 2, CKMB: creatine kinase MB, CVD: cardiovascular disease, DM: diabetes mellitus, ECs: endothelial cells HFpEF: heart failure with preserved ejection fraction, HG: high glucose, MCAO: middle cerebral artery occlusion, I/R: Ischemia-reperfusion, MACCE: major adverse cardiac and cerebrovascular events, PBMNCs: peripheral blood mononuclear cells, PCI: percutaneous coronary intervention, PTEN: phosphatase and tensin homolog, SIRT1: Silent information regulator 1, STEMI: ST-segment elevation myocardial infarction, VSMCs: vascular smooth muscle cells.

Recent data showed that metformin reverted impaired angiogenesis caused by palmitic acid treatment of HSPCs by the miR‑130a-3p/phosphatase and tensin homolog (PTEN)/ phosphoinositide 3-kinases/protein kinase B axis [270] in mouse microvascular ECs by reducing miR-34a-5p and increasing silent information regulator 1 (SIRT1), a miR-34a target [271].

Metformin is also able to reduce oxidative stress, which is a crucial mechanism of cardiac injury caused by ischemia-reperfusion (I/R), a major side effect of the reperfusion treatment of the ischemic heart [272]. This beneficial effect is associated with the regulation of a p53-miR-34a-5p-Bcl-2 pathway axis [273]. Moreover, Zhang et al. demonstrated that metformin prevented hydrogen peroxide (H_2_O_2_)-induced neonatal rat ventricular cell death by reducing miR-1a-3p via AMP phosphorylation and inducing the expression of its target glucose-regulated protein 94, resulting in decreased cell death signaling activation [274, 275]. Recently, it has been reported that metformin exerts an epigenetic effect in T2DM South African patients. Indeed, several DNA regions were differentially methylated and 57 lncRNAs-associated DNA methylation regions were identified in the genomic DNA of metformin-treated T2DM patients.

The expression of lncRNA-H19 is closely related to the progression of cerebral ischemia [276]. Zeng et al., in a stroke mouse model [277], reported that lncRNA-H19 expression was elevated and that it participated in the ischemic stroke-induced oxidative stress injury via inhibition of miR-148a-3p and upregulation of its target Rho associated coiled-coil containing protein kinase 2, which increases oxidative stress [278]. These responses were reduced by metformin treatment that ameliorated stroke-related brain injury [277].

Among the new generation of anti-diabetic drugs, liraglutide, a glucagon-like peptide-1 analog, in diabetic rats improved significantly aortic endothelial dysfunction by downregulating miR-34a-5p and increasing the expression of anti-apoptotic protein B-cell lymphoma 2 (Bcl2) and SIRT1 [279]. Moreover, recently it has been shown that liraglutide therapy increased serum levels of miR-130a-3p, miR-27b-3p, and miR-210-3p in patients with T2DM [280].

Empagliflozin is a novel selective inhibitor of sodium glucose cotransporter 2 associated with benefits on mortality, HF hospitalizations, and quality of life [281]. In a preliminary study with a small group of frail old adults with HF with preserved ejection fraction (HFpEF) and DM patients compared to controls, circulating levels of miR-21 and miR-92 were found to be significantly increased. The levels of these miRNAs decreased after 3-month treatment with empagliflozin, but not in response to metformin or insulin [282].

#### Anti-inflammatory/anti-aggregation drugs and ncRNAs

Acetylsalicylic acid (ASA) is a widely used nonsteroidal anti-inflammatory drug. Low doses of ASA inhibit cyclo-oxygenase-(COX)-1 and oppose platelet aggregation inhibiting the release of thromboxane A2 (TXA2) [283, 284], while higher ASA doses inhibit COX-2 and decrease prostaglandin E levels, inducing antipyretic and pain-relieving effects [285]. Accordingly, low ASA doses are prescribed for primary and secondary CVDs prevention [285–287].

Several reports indicate that ASA treatment increased miRNAs expression and that several miRNAs can be useful biomarkers of ASA resistance. For instance, Guo et al. observed that miR-145-5p levels in PB mononuclear cells of ischemic stroke patients are increased by ASA treatment. Moreover, an ASA-mediated increase of miR-145-5p inhibited CD40 mRNA translation, reducing vascular smooth muscle cell proliferation *in vitro*. The interplay between ASA and miR-145-5p improved also the stability of atherosclerotic plaques.

ASA resistance is linked to the variability of platelet reactivity among cardiovascular patients [288]. MiR-135a-5p and miR-204-5p are correlated with platelet reactivity [289], while lower expression of miR-19b-1-5p is correlated with ASA insensitivity [290, 291]. Moreover, decreased levels of circulating miR-223, which is among the highest expressed miRNAs in platelets, is an independent predictor of responsiveness to antiplatelet therapies in patients suffering from CAD [292].

The P2Y12 inhibitors clopidogrel, prasugrel and ticagrelor are antiplatelet agents alternative to ASA. They are characterized, unlike ASA, by a prolonged presence in the plasma [293] and the combination of clopidogrel and ASA has a potentiated effect in the secondary prevention in patients with T2DM [294]. Moreover, ticagrelor and prasugrel when given in combination with ASA have beneficial effects in patients after MI [295]. The potential of clopidogrel or prasugrel antiplatelet therapy versus ASA therapy on platelet function, fibrin network characteristics, inflammation, and expression of miRNAs was investigated in a clinical trial in T2DM patients [296]. Prasugrel monotherapy in T2DM showed to have the strongest effect on platelet inhibition and in reducing the levels of several miRNAs relevant for platelet function and CVDs (miR-24, miR-191, miR-197, and miR-223) [297, 298]. In addition, reduced levels of miR-197 have been identified as a marker of CVDs in T2DM patients treated with prasugrel [296].

## ncRNAs in coronavirus disease 2019 associated inflammation and cardiovascular damage

### Inflammatory response in coronavirus disease 2019: diabetes as a risk factor

**Fig. 7:**
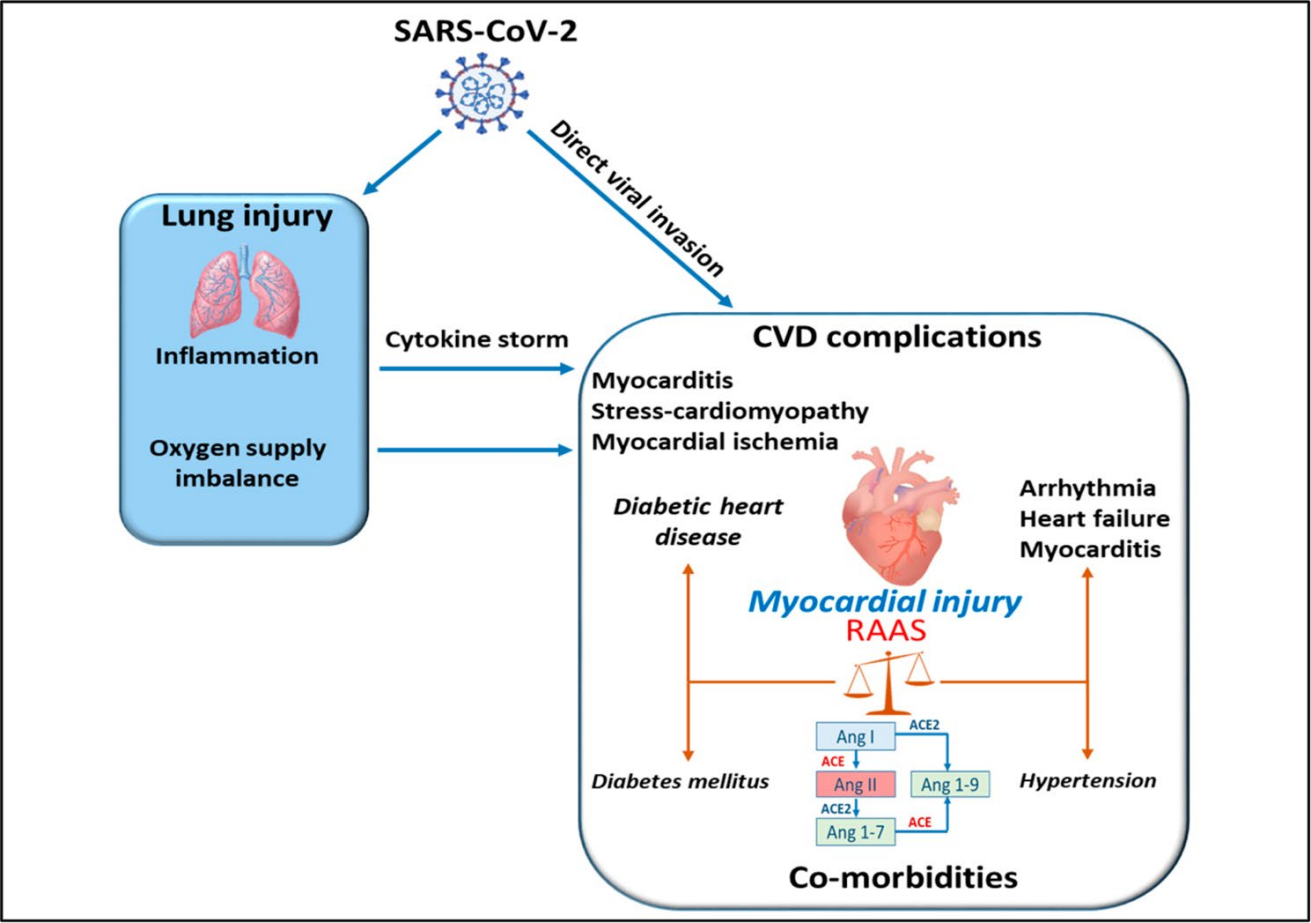
The interplay between SARS-CoV-2 infection, CVDs, and diabetes. SARS-CoV-2 can affect the cardiovascular system directly through invasion of cardiac tissue or, in a more pronounced manner, indirectly, through cytokine storm activation, oxygen supply imbalance, and RAAS dysregulation. DM and hypertension are the most frequent co-morbidities linked to the severity and poor prognosis of cardiovascular outcomes in COVID-19 patients due to imbalanced ACE/ACE2 and RAAS activation. ACE2: ACE2 indicates angiotensin-converting enzyme 2, Ang: angiotensin, CVDs: cardiovascular diseases, RAAS: renin-angiotensin-aldosterone system

Clinical outcomes of COVID-19 are worse in the presence of CVDs and risk factors, such as DM (Fig. 7). Severe COVID-19, DM, and CVDs share a common denominator, an increased inflammatory status, which is also thought to underlie male-biased cardiovascular complications in COVID-19 [299, 300]. Assessing cardiovascular inflammatory status through evaluation of ncRNAs levels appears as a promising strategy to aid in personalized healthcare, prevent fatal cardiovascular damage, and thus improve clinical outcomes of COVID-19 patients. SARS-CoV-2 can affect multiple organ systems directly or indirectly through cytokine storm and the adaptive immune system activation [301, 302], dysregulation of the renin-angiotensin-aldosterone system (RAAS) as a result of virus-mediated suppression of angiotensin-converting enzyme (ACE) 2 expression [303], damage to ECs [304], vascular inflammation and sepsis [305]. Cardiovascular damage is frequently observed in severe COVID-19 patients and it is dictated directly by the amount of viral load or indirectly by the extent of the patient’s immune response. Cardiovascular damage is also dependent on the presence of co-morbidities associated with RAAS deregulation (Fig. 7) [306]. Although severe acute respiratory syndrome coronavirus 2 (SARS-CoV-2) has shown major tropism to the pulmonary cells, recent reports confirmed its presence in myocardial biopsies of COVID-19 patients with myocarditis [307–309]. Cardiac tissues of COVID-19 patients with high viral invasion had increased expression of pro-inflammatory cytokines [307]. However, this finding was not related to the prominent invasion of the cells, thereby indicating that the presence of SARS-CoV-2 in the heart may not fundamentally cause an inflammatory response, which is in line with clinical myocarditis. The long-term consequences of SARS-CoV-2 infection and associated cardiovascular outcomes require advanced examination.

ACE2 is a part of RAAS and plays a critical role in the development of DM, hypertension, and HF. Disturbance of RAAS caused by loss of ACE2 in diabetic patients results in declined vascular function and systolic dysfunction that are dependent on the angiotensin II/AT1 receptor [310]. Since SARS-CoV-2 uses human ACE2 as cell entry receptors and human proteases as entry activators, RAAS is an important component of COVID-19 pathogenesis. Detailed mechanisms of SARS-CoV-2 entry into host cells have been recently reviewed in [311]. Imbalanced ACE/ACE2 and RAAS activation are responsible, at least in part, for COVID-19 progression which can lead to cardiac damage by inducing a hyperinflammatory response characterized by high levels of IL-6, IL-1, and TNF-α [312]. A recent meta-analysis that pooled 76,993 patients from 10 studies has demonstrated that hypertension, renal, liver and cerebrovascular diseases, obesity, smoking history, and current smoking are associated with the severity and poor prognosis of COVID-19 [313]. The mechanisms which increment vulnerability to COVID-19 infection in patients with DM may occur through ACE inhibitors or angiotensin receptor blockers which modulate ACE2 expression or through compromised immunity and elevated furin levels [314]. However, the Council on Hypertension of the European Society of Cardiology emphasized that there is no evidence supporting harmful effect of ACE inhibitors (ACE-I) or angiotensin receptor blockers (ARBs) during the COVID-19 outbreak. The Council strongly advise physicians and patients to continue their anti-hypertensive therapy treatment since there is no scientific or clinical basis for stopping ACEi or ARBs due to Covid-19 ^1^.

A study by Abe et al. has shown that COVID-19 patients with DM had an increased incidence of acute myocarditis, acute HF, acute MI, and atrial fibrillation [315, 316]. Increased serum levels of IL-6, IL-8, and IP-10 were associated with a high mortality rate in COVID-19 patients [315]. The interplay between COVID-19 inflammatory response and DM may help to explain the poor cardiovascular prognosis of patients after SARS-CoV-2 infection, suggesting that interventions targeting innate immune pathways could potentially benefit COVID-19 patients with DM and other cardiovascular risk factors. Overall, applying therapeutic strategies to improve cardiovascular health may improve outcomes in COVID-19 patients, including patients with diabetes [317–319].

### ncRNAs in COVID-19

Several studies highlighted that altered levels of circulating cardiovascular, immunological, and inflammatory ncRNAs could be potential biomarkers of COVID-19 severity and myocardial damage. As an example, Garg et al. observed that inflammation-associated miR-155-5p, profibrogenic miRNA miR-21-5p, and cardiomyocyte-associated miRNAs miR-499-5p and miR-208a-3p were upregulated in the serum of severe COVID-19 patients requiring invasive ventilation in comparison to healthy controls and influenza-acute respiratory distress syndrome patients [320]. Another study showed that circulating myomiRNA miR-133a was upregulated in the plasma of severe compared to mild COVID-19 patients [321]. In particular, miR-133a overexpression was associated with intensive care unit (ICU) mortality within 28 days. Interestingly, myocardial injury correlated with an increase in circulating miR-133a levels [321]. In this setting, the main source of circulating miR-133a is most likely to be the cardiomyocytes, since they express high levels of this miRNA. However, miR-133a is also expressed in other cell types, such as neutrophils [319], that may be contributing sources of plasmatic miR-133a levels, considering that neutrophil degranulation and extravasation play a role in myocyte damage. Indeed, it was observed that circulating levels of miR-133a inversely correlate to neutrophil counts and directly correlate to myeloperoxidase, a neutrophil activation marker associated with cardiovascular outcomes in COVID-19 patients [322]. Other inflammation and cardiovascular-related miRNAs were modulated, e.g. miR-21-5p, miR-142‐3p, and miR‐146a‐5p were downregulated, and miR-15b-5p was upregulated, in red blood cell‐depleted whole blood of severe COVID-19 patients compared to moderate COVID-19 patients.

De Gonzalo-Calvo et al. [323] showed that miRNAs implicated in immune/inflammatory response and coagulation were altered in the plasma of COVID-19 patients. In particular, miR-27a-3p, miR-27b-3p, miR-148a-3p, miR-199a-5p and miR-491-5p were upregulated and miR-16-5p, miR-92a-3p, miR-150-5p, miR-451a and miR-486-5p were downregulated in ICU patients compared to ward patients. Moreover, three miRNAs (miR-148a-3p, miR-451a, and miR-486-5p) discriminated between ICU and ward patients. miR-16-5p, miR-92a-3p, miR-98-5p, miR-132-3p, miR-192-5p, and miR-323a-3p were downregulated between non-survivors and survivors to ICU stay. Two miRNAs (miR-192-5p and miR-323a-3p) distinguished between ICU non-survivors and survivors. Of note, a recent investigation has shown the potential of miRNAs, including miR-145a-5p and miR-181-5p, to define endotypes in critically ill COVID-19 patients [324], which suggests their usefulness in personalized medicine.

Recently, our investigations indicated that decreased plasma levels of miR-144-3p and − 5p were associated with both mortality and disease severity grade in three independent cohorts of hospitalized and non-hospitalized COVID-19 patients [325]. Thus, circulating miR-144 emerges as a noninvasive tool for early risk-based stratification and mortality prediction in COVID-19. This result is noteworthy in light of the reduced plasma levels of miR-144 observed in patients with DM with cardiac dysfunction [223].

Additional investigations have proposed that the disease not only impacts the circulating miRNA profile during the acute phase but also in “long-COVID-19”. Plasma levels miRNAs implicated in diverse immune mechanisms, e.g. miR-9-5p or miR-146a-5p, or miR-221-5p, have been associated with pulmonary dysfunction and radiologic abnormalities after hospital discharge in survivors of severe COVID-19 [326].

Collectively, the results show the association between COVID-19 severity and the alterations in the circulating miRNA profile, which may also contribute to the inflammatory effects of the disease. Indeed, circulating miRNAs could serve as biomarkers for clinical decision-making, but also as targets for developing specific therapies. For instance, Meidert et al. has proposed different EV-derived miRNAs, including let-7e-5p, miR-146a-5p, and miR-3168, as key regulators of signaling pathways implicated in inflammation and immune function in COVID-19 patients [327]. Results from independent publications support the participation of immune- and inflammatory-related miRNAs in the modulation of these mechanisms [328, 329]. In the context of metabolic disease, Wang et al. identified three antiviral miRNAs, miR-7-5p, miR-24-3p, and miR-223-3p, showing anti-inflammatory and cardio-protective roles, and remarkably decreased levels in serum exosomes of patients with DM compared to non-diabetic controls. The altered levels of these exosomal miRNAs in circulating exosomes weakened the inhibition of SARS-CoV-2 replication in patients with DM [49, 330, 331]. Therefore, miRNAs could serve as potential therapeutic candidates to prevent the progression from mild to severe COVID-19 disease in patients with DM by reducing viral replication.

## Conclusions

Although the field of ncRNAs is still largely unexplored, it is now clear that ncRNAs play a crucial role in inflammation and DM. As described in this article, indeed, many ncRNAs are modulated in their expression in DM patients, can be transferred between inflammatory cells and cardiovascular cells inducing changes in their respective functions, representing in many cases viable therapeutic targets. Moreover, ncRNAs can be detected in the circulation representing valid biomarkers for the evolution of the disease.

Limitations to our understanding of ncRNAs in DM and inflammation are still present, due to incomplete sequence annotation (both structural and functional) and only partial conservation (more structural than sequence conservation). Publically available databases are dispersed and each one of them is incomplete in some aspect. In this respect, transcriptomic datasets are often only restricted to poly (A+), thus limiting the information to the mature form of mRNAs and a subset of lncRNAs.

Despite these difficulties, the current knowledge about ncRNAs has already allowed us to unveil many complex networks, shedding light on the multiple interactions governing the inflammatory response in the context of DM, and many more are likely to be identified in the upcoming years. We are beginning to understand that the potential of RNA therapy is enormous and results have started coming (ncRNAs clinical trials, development of RNA-based COVID-19 vaccines). Therefore, we believe that new larger cohort studies and further insights into the mechanisms of action will allow the clinical translation of prevention, prognosis, and cure of DM cardiovascular complications via ncRNA-based control of immune cell regulation.

## Data Availability

Not applicable.

## List of abbreviations

ACE: Angiotensin-converting enzyme
ASA: Acetylsalicylic acid
ASOs: Antisense oligonucleotides
Bcl2: B-cell lymphoma 2
BM: Bone marrow
CAD: Coronary artery disease
Cas9: CRISPR associated protein 9
CASP: Caspase
CCC: Cell-to-cell communication
CircANKRD36: Circular ankyrin repeat domain 36
CircRNA: Circular RNA
CLI: Critical limb ischemia
COVID-19: Coronavirus Disease 2019
COX: Cyclo-oxygenase
CRISPR: Clustered regularly interspaced short palindromic repeat
CVD: Cardiovascular disease
DCs: Dendritic cells
DM: Diabetes mellitus
Dnm3os: Dynamin 3 opposite strand
DRAIR: Diabetes regulated anti-inflammatory RNA
EC: Endothelial cell
ENVIRONAGE: Environmental influences on early ageing
ERK: Extracellular signal-regulated kinase
EVs: Extracellular vesicles
Fox: Forkhead box
GalNAc: N-Acetylgalactosamine
GAS5: Growth arrest specific 5
HF: Heart failure
HFpEF: Heart failure with preserved ejection fraction
HOTAIR: Homeobox transcript antisense RNA
HSPCs: Hematopoietic stem/progenitor cells
HuR: Human Antigen R
I/R: Ischemia-reperfusion
ICAM-1: Intercellular adhesion molecule 1
ICU: Intensive care unit
IFN-γ: Interferon gamma
IL-1β: Interleukin-1β
KCNQ1OT1: Potassium voltage-gated channel subfamily Q member 1 opposite strand/antisense transcript 1
KLF: Krüppel-like factor
LDL: Low-density lipoprotein
LINC01005: Long intergenic non-protein coding RNA 1005
LNA: Locked nucleic acid
lncRNA: Long non-coding RNA
MALAT-1: Metastasis associated lung adenocarcioma transcript-1
MAPK: Mitogen-activated protein kinase
MEK: Mitogen-activated protein kinase kinase
MI: Myocardial infarction
MIAT: Myocardial infarction–associated transcript
miRNA: MicroRNA
mRNA: Messenger RNA
MVs: Microvesicles
ncRNA: Non-coding RNA
NET: Neutrophils extracellular trap
NEXN-AS1: Nexilin F-actin binding protein antisense RNA 1
NF-κB: Nuclear factor kappa B
NK: Natural killer
NLRP3: NOD-, LRR- and pyrin domain-containing protein 3
Ox-LDL: Oxidized low-density lipoprotein
PB: Peripheral blood
piRNA: Pwi-interacting RNA
PTEN: Phosphatase and tensin homolog
PAD: Peripheral arterial disease
RAAS: Renin-angiotensin-aldosterone system
ROS: Reactive oxygen species
rRNA: Ribosomal RNA
SARS-CoV-2: Severe acute respiratory syndrome coronavirus 2
sEV: Small extracellular vesicle
SIRT1: Silent information regulator 1
sncRNA: Small ncRNA
snoRNA: Small nucleolar RNA
snRNA: Small nuclear RNA
STZ: Streptozotocin
T2DM: Type 2 diabetes mellitus
TGF-β: Trasforming growth factor-beta
Th1: T-helper-1
TLR: Toll-like receptor
TNF-α: Tumor necrosis factor-α
T-regs: Regulatory T cells
tRF: TRNA-derived fragment
tRNA: RNA transfer
TXA2: Thromboxane A2
VCAM-1: Vascular cell adhesion molecule 1
VSMC: Vascular smooth muscle cell
